# Abstracts and Gleanings

**Published:** 1882-07-20

**Authors:** 


### ABSTRACTS AND GLEANINGS.

**Pathology and Microscopy.**—In a report on Pathology and Microscopy, read before the North Carolina Medical Society, by H. W. Lilly, M. D., of Fayetteville, N. C., it is remarked :

We are indebted to the London Lancet for the following general resume of recent investigations in Pathology :

The researches of the year which have most attracted attention are those of Pasteur, in France, which have reference to bacterial pathology. He has made extended investigations and experiments in this direction, and together with the contributions of Touissaint, Klebs, Arloing and Thomas, he now includes quite a large number of diseases in that class which is thought to depend upon the existence of special organisms and to which the practice of prophylactic inoculation is applicable. Typhoid fever has for some years been thought to be related in some way to a special organism, and the researches of Pasteur and Klebs have tended to confirm this to a great extent. A great many of the pathological changes which mark this disease are thought by them to bear to this organism a very intimate relation; and the idea is well borne out by the experiments of Branlecht, which were made during an epidemic of typhoid fever, and which resulted in the discovery of an organism in the drinking water very similar in its nature to that noticed by Pasteur and Klebs.

Diphtheria has been produced by Talamon by means of inoculation, the inoculating matter being obtained from the throat, lungs and kidneys of a diphtheritic patient. This shows the existence of a special organism.

Investigations have been made by Cornil and Neisser which show conclusively that leprosy depends upon the presence of a special bacterial organism; and by a close study of the manner and growth of these organisms, the peculiar development of the bacteriæ is thought to account for the strange lesions by which the disease manifests itself.

In syphilis, there has been discovered by Aufrecht a specific micrococcus which is thought to be the pathogenic agent. This view, however, has not been supported. It seems, therefore, that bacterial organisms are not confined exclusively to acute diseases; and their recent detection as morbid agents in the production of a few chronic disorders has given birth to the hope that a great many chronic diseases, enveloped now in mystery, may be traced to a similar origin, and be amenable, therefore, to a moie rational and decided plan of treatment.

So great has been the interest and enthusiasm manifested by investigators in bacterial pathology during the past year, that it has tended to divert their attention from other important subjects.

But in the Annus Medicus we find a condensed account of the labors of pathologists in other directions. It is little more, however, than a mere index to their work.

Among chronic diseases, tuberculosis has been afresh studied in its aspect as an infectious disease by Rindfleisch and Creighton, but with no very important additions to the literature of the subject. Some interesting and rare forms of local tuberculosis of the mouth and throat have been described by Russner and Echhoff, and of the urinary organs by Finne, while the histology of tubercular disease of the testes has been carefully studied by Waldstein. The relation of tuberculosis and scrofula has been the subject of animated discussion in Paris, but, while it directed attention to the subject and stimulated further study of the two kindred diseases, it was productive of no new developments. The study of structural blood-diseases has been mainly confined to their connection with blood formation in bone-marrow.

Lyon has examined the changes in the blood in traumatic anæmia, and the result has established the fact that it constitutes a distinct variety of anæmia, but its exact nature was not satisfactorily determined. Compared with the progress of previous years, very little of importance has been discovered in regard to the localization of nerve centres in the brain. What new facts have been noticed have increased rather than lessened the difficulties which before existed in the correct interpretation of experimental facts.

In diseases of the spinal cord, it has been established by Dreschfield that locomotor ataxia may be due to sclerosis(primary) of the lateral, as well as of the posterior columns. It has heretofore been the accepted idea that the posterior columns alone were involved in this affection. When the lateral columns are the primary seat of disease, the ataxis is accordingly modified in its manifestations.

The discussion regarding the relation existing between syphilis and locomotor ataxia has been continued during the present year by Erb, Gowers and others; and it has been conceded that a syphilitic individual is especially predisposed to ataxic disease.

Brown-Sequard has continued his investigations in regard to the production of paralysis and contractures through the agency of the peripheral nerves, and has clearly demonstrated that mechanical irritation of the medulla oblongata may give rise to pulmonary emphysema through the agency of the pneumogastric nerves.

**Hereditary Alcoholic Appetite.**—A gentleman farmer, living in the northern part of England, was much given to strong drink. He was fond of good living and good company. After his marriage he became more intemperate than before, and at last died a confirmed sot, from paralysis and kidney disease. He left six children, three sons and three daughters, who all in time became drunkards; but the eldest son, born when the habits of his father were not so intemperate, was less addicted to it than the others. The second son, while studying law, fell into every kind of debauchery, finally came to America, and died a sot in a hospital in New York. The third son was brought up for the ministry, and gave much promise of ability and moral worth, but he also fell into dissipated ways; and often went drunk into the pulpit. Expelled from his ministerial office, he lived a short life of poverty and drunkenness, and died in a workhouse. The eldest daughter tapped a barrel of rum in her husband's absence, and was found by him dead under it. The second daughter died in a fit of delirium tremens. The third daughter was not so violent a drunkard as her sisters, but nevertheless got drunk whenever she had an opportunity.

The eldest son succeeded to his father's property, and, while he drank more moderately than his brothers, was still spoken of as intemperate. He died a violent death, leaving two sons and three daughters. His eldest son, after a short life of dissipation, died a drunkard. The second son committed a murder under the influence of drink. The eldest daughter had a delicate constitution, and died in giving birth to her first child. The second daughter died of consumption. The third daughter was confined in an insane asylum soon after her marriage.

It is not too much to say that all this ruinous train of circumstances arose from the condition of drunkenness in one man. It not unfrequently happens that generally temperate parents have an intemperate or an idiotic child. This may result from one or both parents having been under the influence of drink when the child was begotten.

Carpenter mentions a case in which a child begotten when both parents were partially intoxicated (but who were generally temperate) was completely idiotic.

"A child, begotten the night its mother had returned from a ball, when she had indulged in considerable wine, early manifested a strong love for drink, and died a drunkard, although none of her other children were addicted to its use."

If we consider the nature of this appetite, we shall find that to a certain extent it is a natural one. An appetite for a stimulant of some kind has been natural to all men, in all countries and ages. Opium, Indian hemp, tobacco, betel-nut, tea, coffee, and oil, are but different articles used by different people as substitutes for alcohol. The craving for alcohol and the condition of habitual drunkenness are but abnormal and excessive developments of this desire for stimulants.—*Dr. Griswold in Ind. Prac.*

**Mercury Used in Dentistry.**—Dr. Eugene S. Talbot, of Chicago, in American Medical Association, read a paper on "The Injurious Effects of Mercury used as in Dentistry." The paper was confined to the use of amalgam fillings in natural teeth.

There can no longer be doubt that amalgam fillings in teeth will sooner or later produce mercurial poisoning. The dire effects of this metal are not always seen immediately after the fillings are inserted, years sometimes elapsing before the injurious effects were felt and noticed.

The history of two well-marked cases were here given by Dr. Talbot, the persons affected having called upon him for treatment. The amalgam fillings were removed, and gutta percha temporarily substituted, these in turn being replaced with gold, after which all symptoms of mercurial poisoning disappeared. A detailed account of a series of experiments made by him were then presented, the conclusions and results being as follows:

1st. Mercurial vapor is given off from amalgam fillings at all ages and from all varieties, even from fillings sixteen years old, the vaporization being sufficient in quantity to respond to chemical tests.

2d. Minute doses of mercury, if taken internally three times a day, are capable of producing decided effects.

3d. Mercury when inhaled into the lungs is far more active than when taken into the stomach.

4th. If small doses taken into the stomach occasionally are capable of producing marked effects, and the vapor is much more active than the solid preparations of the metal, is it not a necessary consequence that amalgam fillings which are constantly giving off mercurial fumes to be inhaled into the lungs, not a few times daily, but always, without cessation, day or night, is it not a necessary consequence that in many sensitive persons such fillings must produce deleterious effects?

5th. When tons of this material are consumed annually, is it not credible that many constitutions are affected?

6th. Physicians in treating dyspeptics, anemics and persons suffering from nervous debility, would do well to examine the mouths of patients and know if artificial teeth on red rubber or fillings of natural teeth have in their composition mercury or any of its compounds.—*Detroit Clinic.*

**The Autopsy Upon the Body of Guiteau.**—We have the pleasure of presenting to our readers the fullest and most authentic report as yet obtainable of the autopsy held upon the body of Guiteau.

It is already known that the arrangements for the examination were deficient in many respects, owing either to lack of opportunity or of knowledge on the part of those directly responsible. With every wish, no doubt, for the contrary, there was an utter failure to arrange for a thorough and scientific study of the brain. We would not deprecate the value of our correspondents' work, however. All the facts of scientific value, and they are many, that could be gleaned between the tumbling of the brain into a grocer's scales and the final distribution of fragments are presented here by them.

We learn that the membranes were slightly diseased. This disease was of a character which indicated nothing regarding Guiteau's insanity.

As to the brain itself, and its convolutions, there was a high degree of fissural ornamentation, a well-marked symmetry of fissural arrangement on the two hemispheres, and an absence of the confluent fissural type. There was a well-marked fissural and general development of the frontal lobes. These also, we are told, had a peculiar shape. There was no gross evidence of disease anywhere in the brain-tissue. It is not at all likely that the microscope could have revealed anything. Under the unfortunate plan of unsystematically cutting the brain in pieces, hardly anything can be expected from the microscope now.

On the whole, therefore, the evidence gathered by Drs. Morton and Dana shows that the brain had probably no detectable disease, that it had a somewhat peculiar fissural arrangement, and that it was of rather a high type. The cause of Guiteau's morbid mental state lay in structural or chemical deviations too delicate for detection with our present knowledge.—*N. Y. Med. Record.*

**Oxyuris Vermicularis.**—Dr. James P. Kingsley writes about these worms in the St. Louis Medical and Surgical Journal. They may exist in considerable numbers, and for a long time, in a child, without attracting notice by any symptoms of importance. The most frequent symptom is scratching of the anus, especially at night, after the child has become warm in bed, an increased amount of mucus at that time favoring the movements of the worms. There are frequent attempts to evacuate the bowels, which in many cases, results in the discharge of a small quantity of mucus. Often there is such violent straining at stool that the bowel becomes prolapsed. Sometimes the worms migrate into the vagina, and there excite great irritation, inflammation of the vulva and leucorrhœa. In the male they may cause erection and sometimes balanitis, also pain upon micturition and defecation.

Since these parasites inhabit the large bowel only, and usually the lower portion of it, they can readily be removed by the use of proper enemata. The best treatment is the daily injection of two or three ounces of lime water into the rectum, together with the occasional administration of a mild purgative, either a teaspoonful of castor oil, or one grain of calomel rubbed up with five grains of sugar, at bed-time. A solution of common table salt injected daily answers an admirable purpose.

When there is a relaxed condition of the bowel, evidenced by its protrusion when straining at stool, an astringent injection should be used. In such cases he uses the following—

℞ Ferri sulphatis.................................ʒj,
Infus. quassiæ,.................................Oj.
M. Sig. Inject four ounces every morning.

The mother or nurse must be careful to wash away all the parasites she can find in the folds about the anus. The great itching that comes on after the patient has gone to bed may be effectually relieved by an application, to and within the anus, of the following ointment:

℞ Iodoform,...................................ʒ ss,

Ung. zinci. oxid...............................℥ ss.

M. In addition to local treatment, tonics are usually required, more especially in strumous children. The preparations of iron are decidedly beneficial.—*Med. and Surg. Rep.*

STUDENT.—"How is it, Doctor, that I always take cold in my head?" Doctor.—"It is a well-known principle, sir, that a cold is most likely to settle in the weakest part!"

**Dr. Byrd on Surgery.**—The following is an extract from Dr. Byrd's address on Surgery, Excisions of the Alimentary Canal, etc., at Ameaican Medical Association:

The history of excisions of portions of the alimentary canal by the surgeon, dates back but a few years, and may be said to be the result of evolution beginning with McDowell's first ovariotomy. In cases of obstruction from stricture, medicine had failed for ages to afford relief, and surgery offered no hope. Occasionally where the constriction was caused by the strangulation of an extended bowel in hernia, the intestine would slough and be thrown out through an abscess, and nature would form an artificial anus. The great fear of entering the peritoneal cavity deterred the surgeon from hoping for anything better or resorting to any more radical means for the relief of the poor sufferers. Dr. Nicholass Senn, of Milwaukee, in a very able and exhaustive report to the Wisconsin State Society on the recent progress of surgery, says: The results of the cases of excision of the stomach may not seem promising, but when we come to review the earlier history of anatomy

THE PICTURE IS NEARLY AS DARK, and it must be taken into consideration that many of these operations were undertaken after extensive adhesions had formed and neighboring tissues become involved. May we not hope with earlier and more accurate diagnosis that the diseased mass may be removed so as to restore the patient to years of health and usefulness? The details of the technique of the operation are so well described in a report of Dr. F. J. Lutz to the St. Louis Medical Society and published in June, 1882, that I forbear to quote. The remarks of the late Dr. John T. Hodgen relative to the operations were quoted in length. From the cases and the analogous ones which the author has studied he draws the following conclusions:

First—Resections of the small intestine may be done to a considerable extent without interfering in any appreciable degree with digestion.

Second—Practiced under suitable conditions the operation is to be considered perfectly legitimate.

Third—The resection may be performed by bringing the divided ends directly into apposition and closing the abdominal wound, by forming an artificial anus. The second and third procedure expose to less subsequent danger.

Fourth—Resection of fibrous and cicatricial structure which are probably more frequent than is generally supposed may cause a radical cure, and the same is the case with epithelioma. On the contrary, resections of cancerous obstructions gives only temporary relief, and at a greater risk.

Fifth—By proper diet after the operation the risk of fecal extravasation may be reduced to a minimum, and the best diet for this purpose is one containing as little fluid as possible.

Sixth—By introducing liquids per anum, and drink in the same way, water is absorbed as by the mouth and there is no sense of thirst; the flow of intestinal fluids is less considerable and the patient is more comfortable.

My first case was that of a farmer at Seehorn, Ill., aged fiftyfive. For years he had been treated for strangulated inguinal hernia, which could not be reduced

Found him with clammy sweat, almost pulseless and unconscious. Cut for the hernia and found eight inches of the ileum and a piece of omentum the size of my hand gangrenous. The bowel had separated at the junction of gangrenous and living portions, permitting extravasation of fecal matter. The omentum was ligated just above the gangrenous portion and the gangrenous part cut off. The ends of the ligature were left long so as to hang out of the wound. The sound omentum was dropped into the abdomen. The two ends of the bowel were stitched into the abdominal opening so that any fecal matter would be passed to the outside. They resembled to some extent the muzzle of a double-barreled gun presenting at the opening. The opening was left large enough to permit the insertion of the nozzle of a syringe into the abdominal cavity so that it might be washed clear of any bits of fecal matter or inflammatory products. The cavity of the abdomen was syringed out with tepid water, a teaspoonful of table salt and carbolic acid to the gallon, night and morning. Quinine and nourishing diet was ordered liberally. The patient rapidly recovered, and two months later was operated on for the cure of the artificial anus. * * * * * * * * *

Heretofore the closure of the artificial anus in many cases has been looked upon as a very difficult thing to accomplish, but I think the plan devised for its cure will make the cases few indeed where it cannot be done.—*Detroit Clinic.*

**Syphilitic Infection of the Finger by Medical Men.**—Prof. Fessenden N. Otis, M. D., communicates to the Independent Practitioner of March particulars of eight cases of syphilis contracted by physicians in making digital examination of the vaginæ of syphilitic women. The initial lesion of this form of syphilis is described as being uniformly a papule, "coming soon to be of a deep red color, and presenting a superficial abrasion, becoming circular and deeper by a slow molecular necrosis; not by ulceration with formation of pus; the secretion thin and serous, and drying into a scab which is soon displaced by the fluid accumulating underneath." He also remarks "the entire absence of induration; in its place a slight, flat, juicy-looking, boggy swelling, or elevation, about like a small peppermint in size and thickness."

As proof of the efficacy of treatment, which was continued in five of the cases for one and a half to two and a half years, he states that subsequently "eight healthy children have been born, and both they and the parents have continued free from any evidence of syphilis."—*Med. and Surg. Jour.*

**Iodoform in Gastric Ulcer.**—Dr. M. J. Redmond (British Medical Journal) uses iodoform in the treatment of gastric ulcer, one three-grain pill three times a day. The vomiting of blood, which had been persistent, diminished under the influence of this drug, and finally ceased; pain and tenderness decreased, and within a month the patient had entirely recovered.—*Detroit Clin.*

**Treatment of Acute Tonsilitis by Ice and Quinine.**—To conclude our exercises this morning, I will show you a case, not of very great interest itself, but one that is frequently encountered in private practice, and therefore important for you to see.

This man is suffering with violent acute tonsilitis; the tonsils, as you see, are still inflamed; they were enormously swollen. He had, when he came in, eight days ago, considerable fever; the temperature at one time was 103½°, but it did not long remain at this height. The attack began with a chill, soon followed by fever, mucous expectoration, sore throat, but nothing like a diphtheritic deposit upon the surface. Both tonsils were swollen; he had high fever. It was a case of more than average severity, and in such cases the attack usually terminates with suppuration of the tonsils. It is rare that treatment will succeed in making the inflammation subside without it.

We gave him ten grains of quinine daily at first, in a single morning dose; afterwards in divided doses. We allowed him to suck ice freely, and, also, bearing in mind our recent case of parotid swelling, we applied the ice in bags to the outside of the throat, assiduously. This was carried out very effectually; for in place of the profuse suppuration which usually takes place in such cases in the tonsils, it has only been superficial and very slight, and has affected only one tonsil. Therefore we have reason to be pleased with the effects of the ice and quinine treatment in this case. Otherwise nothing locally was done; he used a little water as a gargle but no astringents; we relied solely upon the ice which he sucked and had applied to the angles of the jaw.

Now, gentlemen, this is a frequent disease and is very painful; if suppuration occur it is prolonged. I have called your attention to it solely with regard to its therapeutics; it presents no difficulty in diagnosis, though obscure in its pathology. In severe cases I believe that the treatment followed in this patient promises very satisfactory results.—*Med. Summary.*

**Poisoning by Winslow's Soothing Syrup.**—In the Sanitary News, December 15, 1881, there is a report of another death of a child eight months old, from the administration of a teaspoonful of "Mrs. Winslow's Soothing Syrup," the symptoms of poisoning by morphia being well marked. Analyses of this dangerous nostrum have shown that each ounce of the syrup contains *one grain* of morphia; so the dose, according to the directions on the bottle, for a child eight months old, contained *one-eighth* of a grain of morphia. It is about time that legal proceedings should prohibit the sale of such dangerous compounds, when advertised as inoffensive.—*Eclectic Med. Journal.*

**The Abortive Treatment of Buboes.**—Dr. M. K. Taylor, (American Journal of Medical Sciences, April, 1882), claims good results in the abortive treatment of buboes by injections of carbolic acid. He reports twenty cases in which he obtained remarkable success, and states that within the last seven years he has treated nearly one hundred and fifty cases of various forms of lymphadenitis, arising from specific and non-specific causes; and where he saw the cases before the formation of pus was well established, he had not failed to arrest the process immediately, and allay the pain in a few minutes. His method is to inject from ten to forty minims of a solution of eight or ten grains to the ounce of carbolic acid directly into the interior of the inflamed gland. This treatment is of course not original with Dr. Taylor.—*Chicago Med. Review.*

**Necrosis from Arsenic.**—Dr. Goodwillie, in American Medical Association, (Detroit Clinic.) called attention of the section to cases of necrosis from arsenic, and illustrated them with wax models.

Case 1 shows, by two models, necrosis of lower jaw from each ramus forward. The case before and after, with a new deposit of bone without any deformity. Photograph of the lady also shown.

Case 2. Two models showing a case of poison by arsenic and necrosis of right superior maxillary.

*a.* Showing case one week after removal of necrosed bone. Without, in the least, disturbing the soft tissue. Also showing the formation of new bone.

*b.* The new bone complete and the mouth perfect; and no external deformity.

Case 3. Upper maxillary showing abscesses formed at nearly all the teeth, the result of applying arsenic to destroy sensibility of the dentine before filling the teeth.

The above will serve to show the sad results of the improper use of this powerful agent in devitalizing dental pulps.

**Neuralgia.**—Dr. Reginald G. Alexander, writing in the Lancet, makes the statement that it is now a well established fact that neuralgia is a disease arising from debility, and since it is very often mistaken for rheumatism, gout, spinal irritation, etc., he gives the following diagnostic points by which it can be differentiated: 1. Neuralgia occurs when general debility exists, is increased by fatigue, mental or bodily, but is relieved by food, and sometimes by stimulants. 2. The pain, which is sudden, darting and excruciating, exhibits remarkable intermissions, especially in the early stages of the complaint, and the constitutional disturbance is slight (temp., pulse, etc., frequently normal). 3. It is usually unilateral. 4 As the disease advances, tender spots are formed in the course of the affected nerves. Realizing that debility plays so important a part in this disease, he says, as would be supposed, that the treatment must be directed in every case toward improving the general health. Pure air night and day, great cleanliness and sponging with sea salt and water. Hypodermic injections of morphia give immediate relief and are really curative, since by allaying pain they allow the tonic measures to be carried out.—*Med. and Surg Rep.*

**Oral and Dental Surgery.**—Dr. D. H. Goodwillie, of New York, chairman of the section on oral and dental surgery, at the American Medical Association, spoke of the two divisions in this department of the healing art: 1st, dental art or prosthetic dentistry; 2d, oral surgery.

The first is nearly all of a mechanical nature, while the latter treated of all the diseases of the mouth.

He believed that the teaching of this specialty should be from established chairs in medical colleges, where all students, before graduating, should be examined on the principles and practice of this department. Besides practical instruction should be given in an infirmary or hospital devoted to this class of affections. He gave illustrative cases from his personal experience of diseases of the mouth and associate parts, such as inter-oral extirpation of the maxilla with reproduction of bone and no deformity; inter-nasal extirpation of bones of the nose; a new operation for closure of the hard palate and lip in early infancy; treatment of abscesses of the jaw and neighboring parts, etc. These cases were illustrated by diagrams, instruments, and over twenty models in wax.

He closed by saying that he hoped the time was not far distant when there would be endowed universities where every branch of the healing art and allied sciences would be theoretically and practically taught.—*Detroit Clinic.*

**Vaccination after Small-Pox.**—Dr. C. F. Armstrong, of Corunna, Mich., writes:

"I had small-pox in 1856, treated by Prof. Sagar, of Michigan University. A few days since, I was vaccinated by Dr. H. B. Shank, of Lansing, and have as nice a vaccine pock as any one would care to see. I have treated many cases of small-pox in hospital and camp, also in private practice without taking the disease."—*N. Y. Med. Record.*

Two physicians applied a galvanic battery to a colored boy in Richmond, Va., who was said to have been struck dumb for lying to his mother, and he quickly began to talk, in the most distinct and emphatic manner.—*Ex.*

**Prescription for Membranous Dysmenorrhœa.**—Dr. Wm. H. Mussey, of Cincinnati, Ohio, in the Transactions of the Ohio Medical Society, 1879, gives the following prescription for membranous dysmenorrhœa, which we have once before published, but which we are requested to republish:

℞ Pulveris guiaci resinæ }

Terebenthinæ canadensis } .................aa ℥j.

Olei sassafras..............................f. ʒij.

Alcoholis...................................f. ℥viij.

M. Macerate for seven days and strain.

Then add:
Hydrargyri chloridi corrosivi.................gr. x.

Sig. Take twenty drops in wine or sweetened water, night and morning.— *Va. Med. Monthly.*

